# Biomechanical comparison of innovative suture techniques for repair of Achilles tendon rupture in a porcine flexor tendon model

**DOI:** 10.1007/s00402-026-06508-9

**Published:** 2026-09-18

**Authors:** Lasse Weigand, Benjamin Liebrand, Sophia Scheible, Rainer Meffert, Stefanie Hoelscher-Doht

**Affiliations:** Department for Trauma, Hand, Plastic and Reconstructive Surgery, University Hospital Würzburg, Würzburg, Germany

**Keywords:** Biomechanics, Achilles tendon, Tendon surgery, PARS, Pretension, Bunnell

## Abstract

**Introduction:**

In recent years, evidence-based progressive rehabilitation protocols following the surgical treatment of Achilles tendon rupture have been shown to be safe and appear superior to more restrictive treatment regimens. These protocols mainly rely on early mobilization and weight bearing. To enable this during postoperative rehabilitation, a biomechanically stable surgical treatment must be ensured. Different solutions for suturing ruptured Achilles tendons are currently favored by orthopedic surgeons and have been investigated in the past, including promising further developments of traditional approaches such as the combination of pretension and the Bunnell technique, as well as minimally invasive or percutaneous techniques using systems like the Arthrex PARS. However, the best solution in this regard remains unclear.

**Methods:**

We analyzed the biomechanical stability of four different suture techniques using porcine deep flexor tendons as a biomechanical model for human Achilles tendons. The tendons were harvested, prepared and sutured according to their respective group (A: Bunnell, B: Bunnell + pretension, C: PARS-to-PARS, D: PARS + anchor). The four groups (*n* = 12) underwent progressive, cyclic, uniaxial tensile loading in the biomechanical testing machine (ZwickRoell Z020).

**Results:**

Group D (PARS + anchor) showed the highest maximum force at ultimate failure, resisting statistically significant higher loads compared to all the other groups. Pretension led to a non-significant increase in maximum force in the Bunnell groups. This was the case for the force necessary to create a 5 mm gap as well, while the force necessary to create the initial gap was equal in the PARS-to-PARS and pretensioned Bunnell group, both proving superior to the Bunnell group without pretension. Group D consistently resisted statistically significant higher forces in comparison to the PARS-to-PARS group for the initial gap, the 5 mm gap and until failure. This was generally valid for the number of cycles necessary to reach the initial gap, the 5 mm gap and the point of failure, as well.

**Conclusions:**

Considering the superiority over both Bunnell techniques during the biomechanical testing in this experimental model despite its limitations in transferability, using the PARS-to-PARS technique in suturing ruptured Achilles tendons seems to be a valid alternative from a biomechanical point of view. We hypothesize that its higher resistance to tension forces found in this study could provide the potential to facilitate more progressive postoperative treatment protocols reflected by earlier weight bearing, which could enable improved rehabilitation outcomes and should be investigated in future studies. While forces acting on the human Achilles tendon during walking in an ankle foot orthosis (AFO) with a lifted heel are estimated at approximately 200 N, our results indicate resistance within a similar force range for the PARS technique. We hypothesize that this technique may contribute to a reduced risk of rerupture, which should be investigated further. Using suture anchors increases the stress resistance of this system further, which makes it a viable alternative not only in distal ruptures. When an open approach is desired, applying pretension can improve the stress resistance of a traditional approach like the Bunnell method, so surgeons should consider this adaptation in such cases.

## Introduction

Achilles tendon rupture (ATR) is one of the most frequent tendon injuries with incidence rate reports of around 11 to 55 per 100,000 person-years in the literature with variances depending on factors like region, sex or study population [1–3]. When reviewing all cases, the typical patient is a middle-aged, male recreational athlete. The incidence peak is at around 44 years, the male-to-female ratio is around 3:1 [4] and a majority of over 80% of the injuries are sustained during recreational or amateur sports [1]. In recent decades, ATR incidence was continuously rising with a particular increase in the age group of 40–59 years and is predicted to rise further in light of aging populations and growing popularity of recreational sports in older age groups [4]. When treating patients with this type of injury, orthopedic surgeons generally can offer different nonoperative as well as operative treatment strategies. Parameters like topography of the rupture, gap size and individual patient characteristics can help surgeons decide whether to operate on this type of injury or treat conservatively. In many cases, there is no clear consensus in the literature regarding the optimal choice of treatment, surgical strategy and posttraumatic or postoperative rehabilitation protocol, respectively [5].

### Indications for surgery and surgical approach

To the best of the authors’ knowledge there are no current, evidence-based and comprehensive guidelines for the treatment of ATR by any major national or international orthopedic association at the time of publication. Even though Chiodo et al. together with the American Academy of Orthopaedic Surgeons (AAOS) provided valuable clinical recommendations for treatment of ATR patients in 2010 [6], their clinical practice guideline summary naturally cannot reflect developments in recent years. In 2026, Feng et al. published current recommendations for treatment of acute ATR from an international consortium of scientists, surgeons and experts in the field as clinical practice guidelines independently of national medical associations. The authors view surgical treatment as indicated for “active, high demand patients or athletes” with an age of ≥ 18 years, positive clinical tests with well-preserved soft tissue and the confirmation of primary acute complete rupture by radiological assessment. The level of recommendation is reported as strong, the level of evidence as weak by the authors [5]. Further considerations by orthopedic surgeons concern the exact location and morphology of the rupture. In proximal ruptures in the myotendinous or musculotendinous junction (MTJ) nonoperative treatment is often preferred due to the lack of support of sutures in the muscular region. In a recent systematic review by Lameire et al., the authors attributed this preference to an apparent good posttraumatic strength and return to activity despite the limitations in the literature [7]. Furthermore, the inability of fully closing the rupture gap with the tendon ends in plantarflexion of the ankle is often regarded as an indication for surgery [8].

Even though in order to achieve an improvement of both ultimate tensile force and resistance to tendon end gapping, the application of additional pretension is an approach used by many orthopedic surgeons in tendon reconstruction, it was not biomechanically investigated with respect to Achilles Tendon reconstruction, extensively. However, there have been some studies reporting promising biomechanical results in the past [9, 10]. In order to find another way to improve traditional open surgical techniques, following the first description of a percutaneous technique for reconstruction of ruptured Achilles Tendons by Ma and Griffith in 1977 [11], the investigation and clinical appliance of minimally invasive techniques gained increasing popularity. These are often associated with lower rates of perioperative complications while biomechanical stability has often been a concern in the past. Addressing those concerns, in a recent meta-analysis by Attia et al. the rerupture rate and functional results were found to be approximately equivalent while minimally invasive techniques were associated with shorter intraoperative time, lower rates of perioperative wound infections and a higher risk of sural nerve lesions compared to open techniques [12]. The Percutaneous Achilles Tendon Repair System (PARS) is available since 2010 as a minimally invasive surgical approach and showed promising clinical results in a first study published by Hsu et al. in 2015 [13] as well as in later clinical studies [13, 14], whereas different in-vitro biomechanical studies demonstrated inconsistent findings in the past, in some cases with superiority [15, 16], in others with inferiority [17, 18] of that system compared with open techniques.

### Postoperative rehabilitation protocols

Even though there is no clear scientific consensus on how exactly postoperative treatment should be performed and despite the subsequently high variability of those rehabilitation protocols between orthopedic departments and surgeons worldwide, they usually share common features. Generally speaking, immediately after surgery there is usually a varying period of restricted range of motion (ROM) of the ankle, where the talocrural joint is externally being immobilized in plantarflexion of varying extent by casts or orthoses. At the same time, patients are commonly supposed to avoid loading the ankle immediately after surgery. This is typically followed by a varying period of increasing ROM towards dorsiflexion, orthoses with progressively neutralizing talocrural angle or shoes with decreasing heel wedges and progressive load bearing. The healing progress is usually being monitored by clinical and sometimes radiological assessment. Complete return to sports is the most common final objective in those protocols, which is usually supposed to be achieved after several months. The individual duration and exact design of those phases is highly dependent on the surgeon’s experience and not necessarily always backed by scientific evidence, even though there have been successful efforts to develop and promote evidence based postoperative treatment protocols in recent years [19]. In the past, postoperative rehabilitation often had a tendency towards long periods of immobilization and non-weight-bearing. However, this was followed by an ongoing trend from those rather restrictive posttraumatic or postoperative treatment towards more progressive rehabilitation protocols with a tendency of shorter periods of immobilization and earlier weight bearing [20]. In the scientific literature, there are indications of these progressive protocols being safe and considering the postoperative outcome probably even superior over more restrictive ones, which Massen et al. were able to show in a 2022 meta-analysis [21]. Among others, possibly the complication that is being regarded as the most critical one following the surgical reconstruction of the Achilles Tendon is rerupture. Rerupture can be considered as a result from a mismatch between biomechanical stress and the stability of the reconstructed tendon at a certain point in its healing process. Therefore, the biomechanical stability of the reconstructed tendon is critical for ATR patients and orthopedic surgeons when aiming to realize progressive postoperative treatment protocols.

The goal of this study was to contribute to the investigation for the best possible treatment of ATR patients. We hypothesized that both the application of pretension as a modification of the traditional Bunnell suture technique as well as the minimally invasive PARS technique are biomechanically superior over the traditional open Bunnell technique and therefore suitable for the realization of more progressive postoperative treatment protocols.

## Methods

### Model and specimen preparation

For this study, a porcine deep flexor tendon was chosen as an established model for human Achilles Tendons in various biomechanical test setups [22, 23]. We harvested the necessary tendons using 48 porcine hind limbs that were obtained as slaughter by-products from a regional slaughterhouse (Friedrich Neckermann GmbH EG Schlacht-und Zerlegebetrieb, Aub, Germany). As we only used post‑mortem tissues obtained from vertebrate animals slaughtered for food production, no animals were killed for research purposes and no experimental procedures were performed on the animals prior to slaughter, the study design was in accordance with the German Animal Welfare Act (TierSchG) and did not require additional approval by local authorities.

**Fig. 1:**
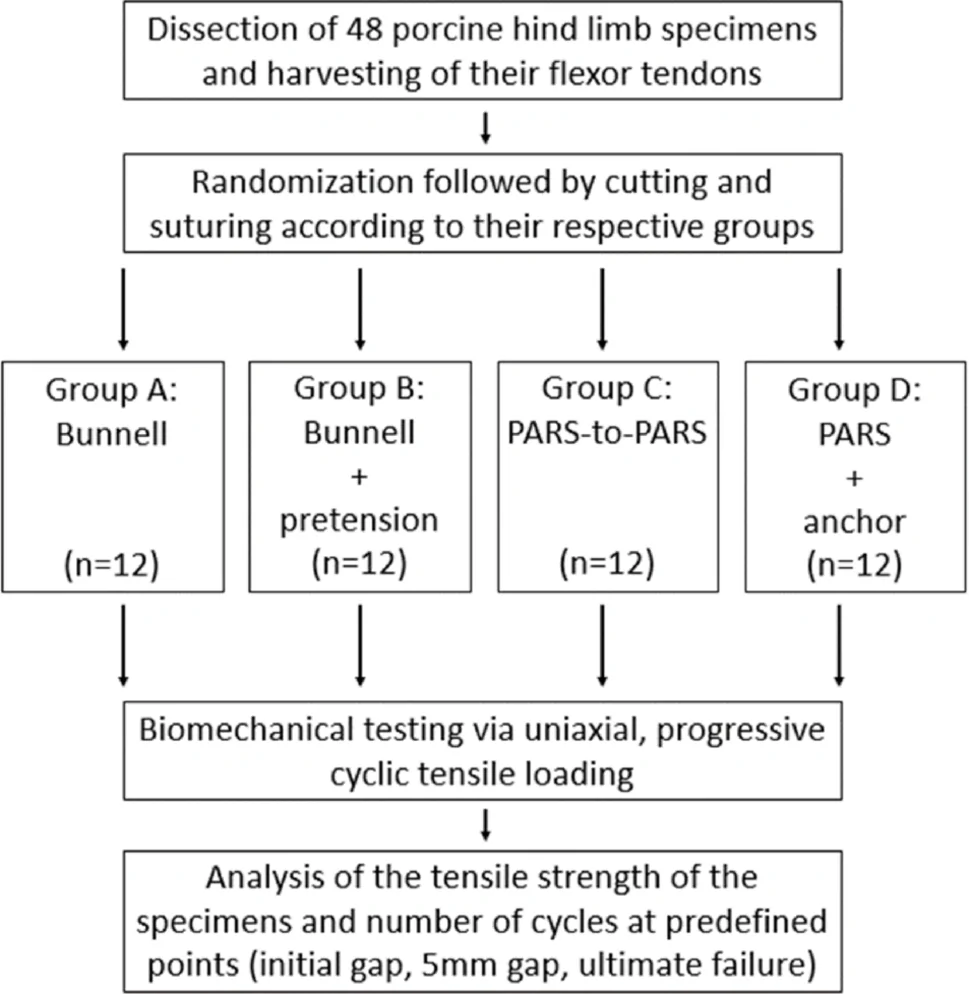
Study design

**Fig. 2:**
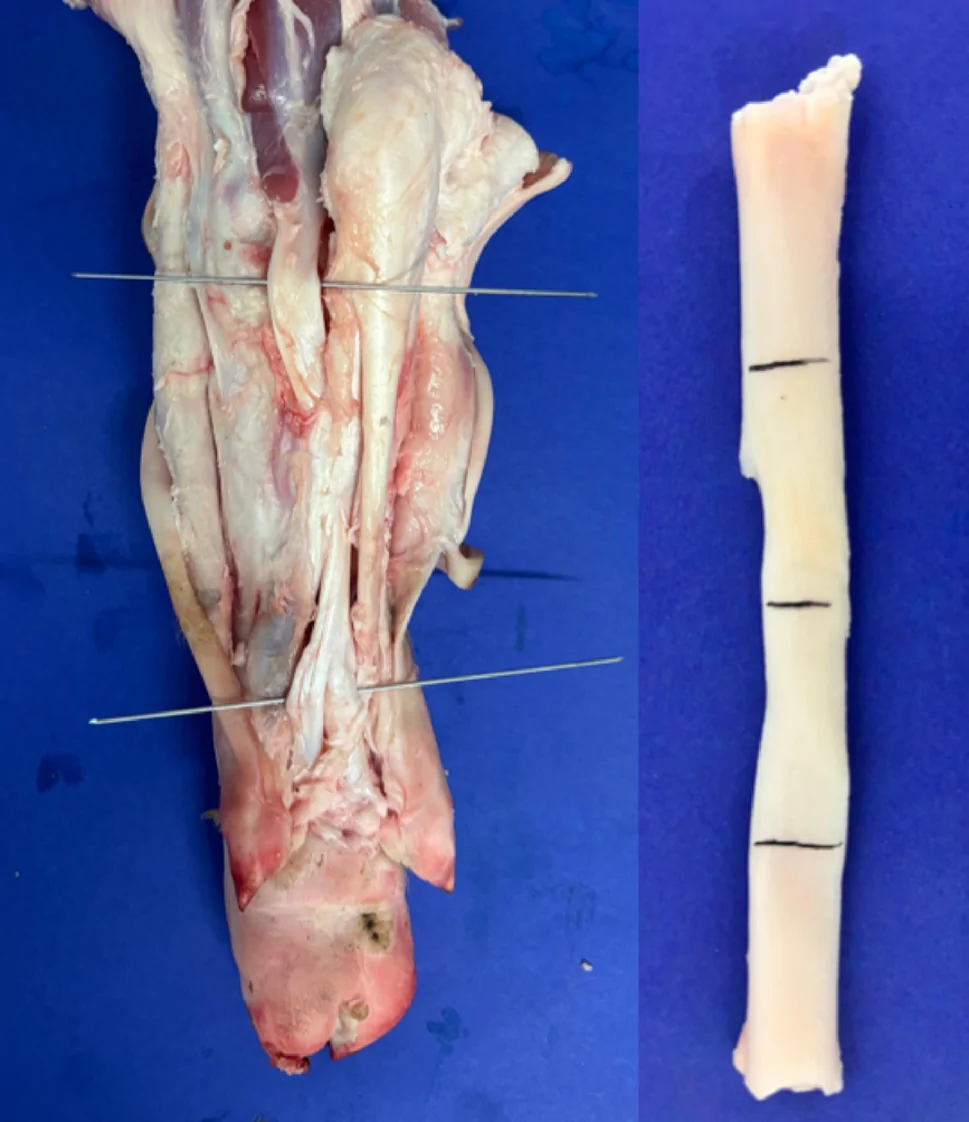
Specimen dissection and preparation

After dissecting the specimen performing a longitudinal skin incision followed by blunt preparation, we identified the deep flexor tendon over the course of its proximal musculotendinous junction until its distal tendinous division. After their explantation, the tendons were cut to a length of 120 mm within their central part, measured and investigated for macroscopic lesions. The tendons were then randomly assigned to 4 groups (*n* = 12), prepared and sutured according to their respective group (A: Bunnell; B: Bunnell + pretension; C: PARS-to-PARS; D: PARS + anchor). After harvesting, the tendons were frozen at -20 °C and thawed for 2 h at 21 °C room temperature prior to testing. During testing, all tendons were hydrated using saline spray to prevent desiccation (see Figs. 1 and 2).

**Fig. 3:**
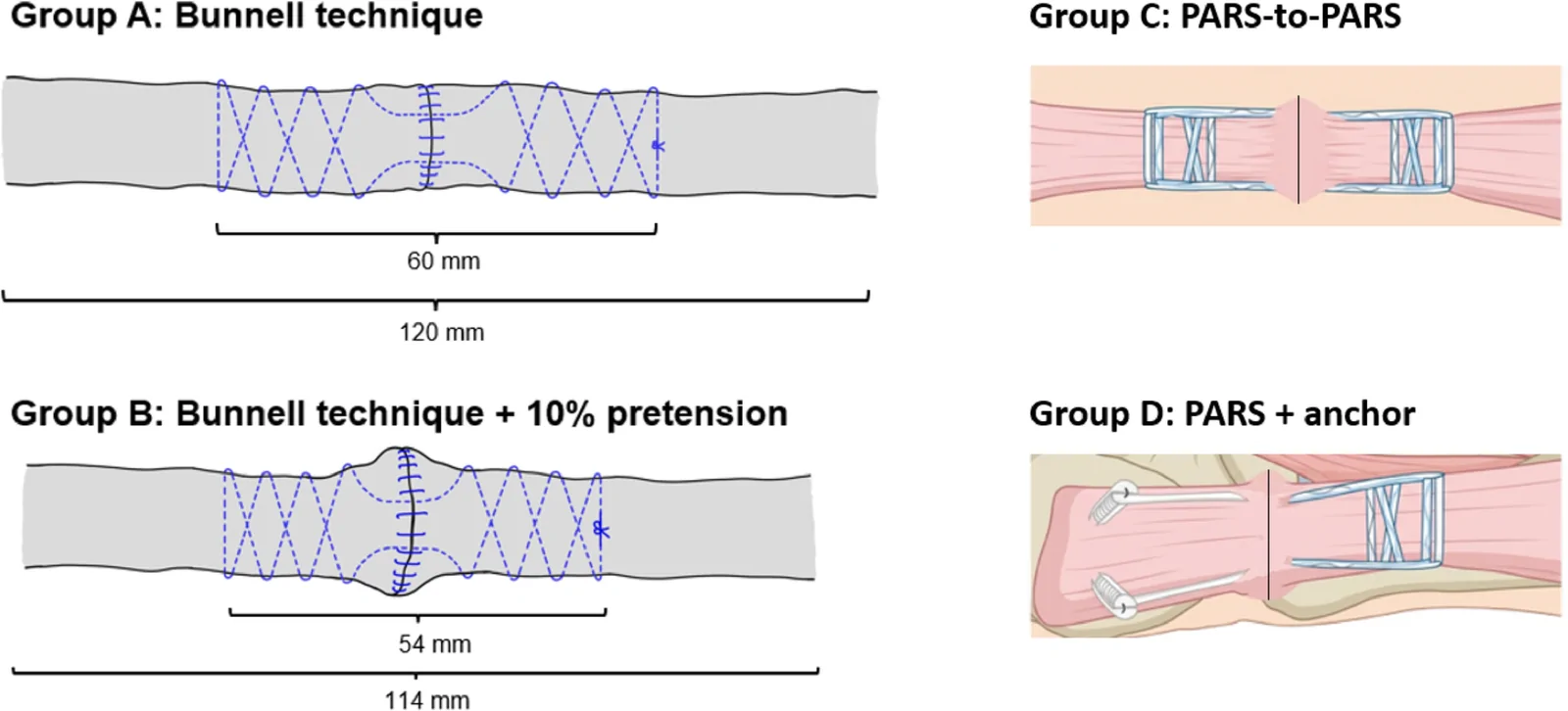
Schematic suture technique in the analyzed groups (C and D made by Biorender)

In group A the specimens were cut transversally in their middle, leaving 60 mm on both sides and repaired afterwards via the Bunnell technique, using a 2 strand Bunnell monofilament slowly absorbable core suture (PDS II 0, Ethicon Inc., New Jersey, USA) and an additional monofilament epitendinous suture (PDS Plus 4 − 0, Ethicon Inc., New Jersey, USA) as shown schematically in Fig. 3. The Bunnell suture was performed over a length of 30 mm on each tendon end, leading to a sutured length of 60 mm of the reconstructed tendon. Tendons in group B were prepared the same way but with an additional longitudinal pulling of the core suture until an evenly distributed shortening of 6 mm of the reconstructed tendon was achieved, leading to a pretension of 10% which was validated by another length measurement and completed by the same epitendinous suture afterwards. Specimens in group C were prepared by using the PARS-to-PARS technique (Arthrex, Naples, USA) following the instructions from the manufacturer [24]. Both ends of the transected tendons were inserted one after another between the inner arms of the PARS jig and sutured (#2 FiberWire Sutures, Arthrex, Naples, USA) the way it was predetermined by the holes in the outer arms of the PARS jig. After removal of the jig, the suture ends were surgically knotted so a continuity of both tendon parts could be achieved as shown in Fig. 3. Group D was prepared accordingly to group C but only using the suture tape in the proximal part of the transected tendon. The distal part was prepared by leading the suture ends of the proximal part longitudinally and centrally through the tendon and fixated with the Achilles Midsubstance SpeedBridge implant system (Arthrex, Naples, USA) to the calcaneus of the respective hindlimb. In order to achieve this, the calcaneus was explanted and cut through transversally in a standardized way 50 mm anterior of its posterior end using a handheld electric saw. Afterwards, two 3,5 mm drill holes were inserted in a 45-degree angle in the posterior end of the calcaneal specimen followed by a 4,75 mm tap. The sutures were then secured in the prepared holes using two suture anchors (SwiveLock^®^ Anchors, Arthrex, Naples, USA) as shown in Fig. 3.

### Biomechanical testing

In order to evaluate the biomechanical properties of the prepared specimen a mechanical testing machine (Z020, Zwick/Roell GmbH; Ulm, Germany) was used together with the software TestXpert III provided by the manufacturer. All tendons were tested in a uniaxial tensile test setup. Both ends of the respective specimens were fixated in stainless steel clamps with a standardized distance of 70 mm between the clamps. The specimens were then tested using a cyclic protocol with progressive loading, starting with a preload of 5 N, followed by 10 cycles per loading level. The levels were set at linear increments of 10 N until total failure of the construct. The speed of the moving traverse was set at 20 mm/min. In order to generate a load-displacement curve, the load and displacement at each time point were recorded by the machine and then analyzed using the testing software. Furthermore, all tests were recorded using a 48MP sensor camera (iPhone 15, Apple Inc., Cupertino, CA, USA) with a frame rate of 60 frames per second rigidly mounted on a tripod in combination with a metric reference positioned in the testing plane in order to identify the formation of an initial gap as well as a 5 mm gap between the tendon ends. The failure mechanism of the construct was recorded and analyzed this way as well.

### Statistical analysis

Statistical analysis was conducted using IBM SPSS Statistics for Windows, version 28.0 (IBM Corp., Armonk, NY, USA) and in consultation with a clinical statistician. Data were tested for normal distribution using the Shapiro-Wilk test and for homogeneity of variance using Levene’s test. Due to unequal variances, a Welch one-way ANOVA was performed followed by pairwise comparisons of the groups using Games-Howell post-hoc tests. The level of statistical significance was set at *p* < 0.05 for all comparisons. Graphical presentation of the results was realized using OriginPro, version 2021b (OriginLab Corp., Northampton, MA, USA).

## Results

**Fig. 4:**
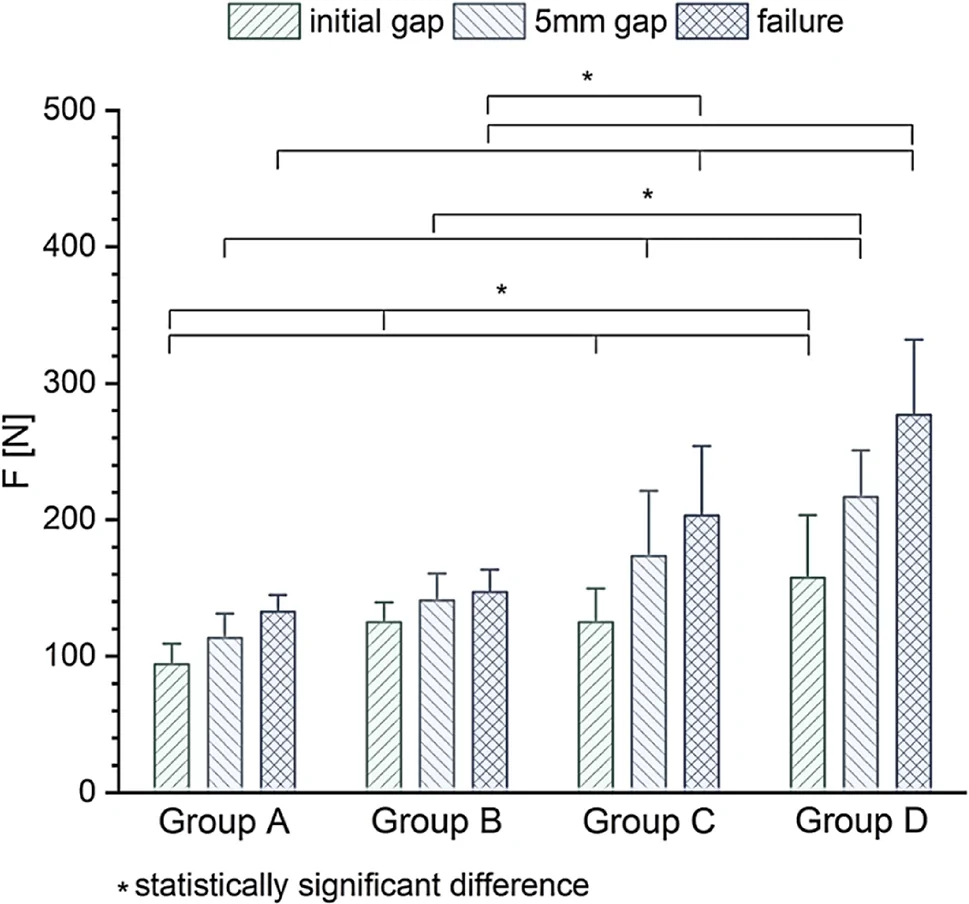
Tensile forces measured for the four analyzed groups at the predetermined points of interest

Group D exhibited the highest mean tensile force at formation of the initial gap between the reconstructed tendon ends, at creation of a 5 mm gap and at ultimate failure of the specimens. The second highest tensile forces were observed for group C but with an equal value as group B for F_initial gap_. The lowest tensile forces at all three points of interest were achieved by group A. Most differences between the four groups were statistically significant, as shown in Fig. 4. This general inter-group ranking was equally valid for the mean number of cycles necessary to reach those predetermined points of interest (see also Tables 1 and 2).

**Table 1:**
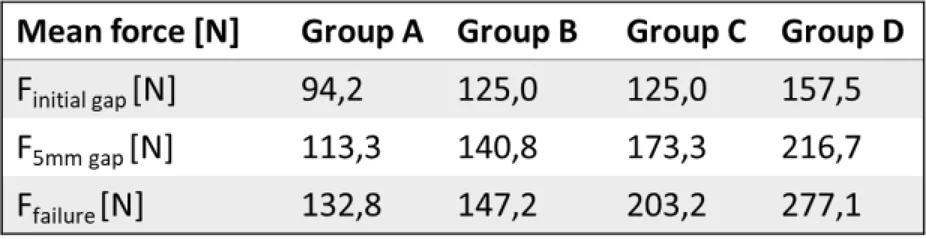
Mean force at predetermined points of interest

**Table 2:**
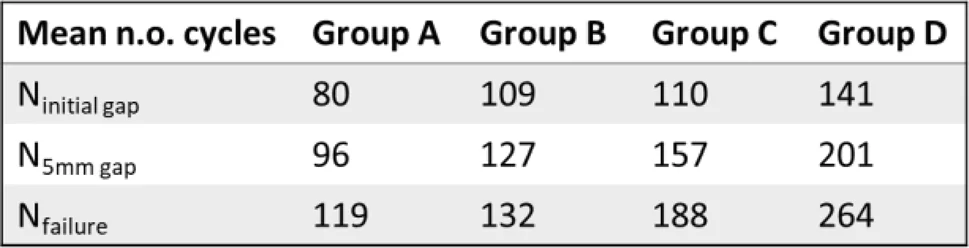
Mean number of necessary cycles to reach the predetermined points of interest

The failure mechanisms differed between the four groups. While suture rupture was the predominant mechanism for almost all specimens of both Bunnell groups, specimens of the PARS groups showed a more diverse pattern. In group C, we observed an equal mixture of suture rupture, suture pullout and a combination of both mechanisms. Reconstructed tendons in group D on the other hand mostly failed by suture pullout as well as in three cases by anchor pullout. Considering the tendon characteristics particularly for the pretensioned Bunnell-reconstructed specimens of group B, we observed a mean shortening from 120,3 mm total tendon length before reconstruction to 113,2 mm after reconstruction (compared to 120,5 mm and 119,3 mm for group A, respectively). This 7,1 mm shortening of the 60 mm reconstructed tendon length reflects a pretension of 11,8%. The mean highest cross-sectional area of group B tendons was increased by 138% to 100,7mm^2^, whereas for group A it was only increased by 78,4% to 72,6mm^2^, respectively.

## Discussion

The observed increase in tensile strength of those tendons undergoing an additional pretension of 10% aligns with the literature. Jordan et al. found a comparable increase in a 2015 study in a similar test set-up [9]. While our goal was to generate a 10% pretension using simple tools like a ruler, the actually achieved mean pretension was 11,8% which we consider very close to the objective and therefore as sufficiently accurate for intraoperative use. There are approaches to standardize pretension for tendon repair aiming for force instead of distance, as well and this might probably be more accurate from a biomechanical point of view. Nevertheless, we regard our method as more realistic for intraoperative use because we consider measuring a decreased reconstructed tendon length with a simple ruler as technically easier and more practical compared to using a tensiometer during surgery, while at the same time being able to standardize the pretension process which traditionally is dominated by the surgeon’s experience. On the other hand, pretension always needs to be balanced and should not be applied excessively in order to prevent restrictions in range of motion, differences to the contralateral side and local tissue problems like reduced peritendinous gliding properties due to the consequently increased cross-sectional area, that we could observe in our study for group B, as well. In our study, the effect of pretension was more relevant for both the creation of the initial gap and of the 5 mm gap, while the ultimate tensile strength of the tendons seemed to be influenced more by the suture configuration. Furthermore, additional application of pretension does not seem to increase ultimate strength and resistance to gapping linearly, as Wu et al. in a 2012 study described the most relevant increase to be found already in 10% pretensioned porcine flexor tendons and only a minor additional biomechanical improvement by 20% pretension [10]. In cases where open surgery is indicated for ATR patients, we recommend considering the application of a moderate pretension of 10% as it can possibly provide a technically easy way to improve the biomechanical stability of the reconstructed Achilles Tendon while limiting the disadvantages of an excessive pretension.

Considering the results obtained for groups C and D, we could observe tensile strengths that were superior to the traditional open reconstruction with the Bunnell technique. The PARS technique is already in clinical use for several years and reportedly successful with respect to the clinical outcome of ATR patients [13]. From a biomechanical perspective, our findings support its status as a valid alternative for surgical treatment of this patient group. While the use of suture anchors in tendon surgery is typically mostly being considered as valuable for ruptures in the osteotendinous insertion zone, in our study we found the tensile strength to be highest for group D. The axial tensile loading in specimens reconstructed with the combination of PARS and suture anchors was distributed differently compared to their PARS-to-PARS or Bunnell counterparts of the other groups as the force was acting not on the distal tendon but along the Sutures running longitudinally through its core as well as on their anchor-augmented insertion in the calcaneal bone as shown in Fig. 3. Nevertheless, following our results, we regard the PARS + anchor technique as a valid alternative not only in very distal ruptures but also more proximally in the midsubstance or mid-section, where most of these injuries occur [25]. By distributing the acting forces through the sutures to the anchors in the calcaneal tuberosity, this approach offers high stability of the suture and good adaptation of the tendon ends, even when tendon quality is poor, compared to purely intratendinous suturing techniques. Compared with open surgery, minimally invasive techniques in Achilles Tendon surgery offer certain general advantages like lower wound infection rates [12], so surgical treatment of ATR patients with the PARS technique might combine those properties with a higher biomechanical stability.

Even though the porcine model used in this study can provide valuable insights regarding the effect of the different suture techniques on the biomechanical stability of the reconstructed tendons, it is subject to several limitations. From a biomechanical perspective, the complexity of an acutely ruptured human Achilles Tendon typically resulting from axial tensile overload certainly is not fully reflected by a model, where the rupture zone is created by transverse dissection. Furthermore, the soft tissue surrounding the tendon may influence its resistance to biomechanical stress, so a direct transfer of absolute force values to in-vivo conditions from testing an isolated tendon model should not be assumed. Nevertheless, our biomechanical model allowed for a very standardized test set-up and homogenous biomechanical properties of the specimens before reconstruction, which potentially strengthens reproducibility of the obtained results. In comparison with in-vivo study designs, another important limitation is the lack of biological healing. As tendon healing cannot occur in this exvivo model, no conclusions can be drawn regarding their biomechanical properties at different postoperative time points. As our study only reflects the biomechanical status at the time of reconstruction, future longitudinal in-vivo study approaches are needed to assess changes in biomechanical stability of the surgically reconstructed tendons over time.

The tensile force acting on the Achilles Tendon seems to be reflected by a wide range of absolute values reported in the literature. Arndt et al. conducted in vivo human optic fibre measurements of the Achilles Tendon force analyzing varying muscular contraction intensities and joint angles in 1998 [26] and found higher forces even for low intensity contractions compared to the results for the reconstructed porcine flexor tendons in our study. Nevertheless, we regard them as clinically relevant considering the special situation during postoperative treatment of this injury. In a 2001 study by Akizuki et al. [27] the plantarflexion torque was estimated on the basis of EMG activity during full weight bearing walking in different ankle foot orthosis (AFO) conditions and found a progressive decrease in torque with immobilization in increasing plantarflexion, subsequently leading to an estimated Achilles tendon force of 191 N for a 1-inch heel lift, which is in the range of the forces observed for our study specimens. Fröberg et al. on the other hand found an increasing mean peak force in the Achilles tendon with increasing plantarflexion when performing optic fiber force measurements and concluded that weightbearing with a ROM restriction to no more dorsiflexion than 20 degrees of plantarflexion may not unload the tendon postoperatively as expected compared to barefoot walking [28]. The question which degree of postoperative restriction in range of motion and load bearing at which point of time after surgery is the most beneficial for tendon healing has been controversially debated since. Despite the natural limitations of transferability due to the model and experimental approach as well as the ongoing scientific discourse in this matter, our results suggest a shift achieved by the investigated techniques towards a target range of postoperative biomechanical stability potentially allowing for earlier weight-bearing in AFOs and earlier reduction of ROM restrictions. Particularly the PARS technique seems to be able to meet the biomechanically higher requirements of these progressive rehabilitation protocols on the reconstructed tendon.

## Conclusion

We conclude that in surgical reconstruction of ruptured Achilles tendons both the application of pretension and the use of the PARS technique can potentially withstand tensile forces within the target range of immediate postoperative biomechanical stability for walking in AFOs, as estimated in the literature. We hypothesize that this higher biomechanical stability compared to the traditional Bunnell technique has the potential to facilitate more progressive postoperative treatment protocols after reconstruction of acute Achilles Tendon rupture as reflected in earlier weight bearing, which could enable better rehabilitation outcomes, potentially contribute to a reduced risk of rerupture and should be investigated in future studies, including more complex experimental models and clinical trials.

## Data Availability

The raw data supporting the findings of this study are available from the corresponding author upon reasonable request.
